# Fibroblast Growth Factor Receptor-2 Contributes to the Basic Fibroblast Growth Factor-Induced Neuronal Differentiation in Canine Bone Marrow Stromal Cells via Phosphoinositide 3-Kinase/Akt Signaling Pathway

**DOI:** 10.1371/journal.pone.0141581

**Published:** 2015-11-02

**Authors:** Rei Nakano, Kazuya Edamura, Tomohiro Nakayama, Takanori Narita, Ken Okabayashi, Hiroshi Sugiya

**Affiliations:** 1 Laboratory of Veterinary Surgery, Department of Veterinary Medicine, College of Bioresource Sciences, Nihon University, Kameino, Fujisawa, Kanagawa, Japan; 2 Laboratory of Veterinary Biochemistry, Department of Veterinary Medicine, College of Bioresource Sciences, Nihon University, Kameino, Fujisawa, Kanagawa, Japan; 3 Laboratory of Veterinary Radiology, Department of Veterinary Medicine, College of Bioresource Sciences, Nihon University, Kameino, Fujisawa, Kanagawa, Japan; Temple University School of Medicine, UNITED STATES

## Abstract

Bone marrow stromal cells (BMSCs) are considered as candidates for regenerative therapy and a useful model for studying neuronal differentiation. The role of basic fibroblast growth factor (bFGF) in neuronal differentiation has been previously studied; however, the signaling pathway involved in this process remains poorly understood. In this study, we investigated the signaling pathway in the bFGF-induced neuronal differentiation of canine BMSCs. bFGF induced the mRNA expression of the neuron marker, microtubule associated protein-2 (*MAP2*) and the neuron-like morphological change in canine BMSCs. In the presence of inhibitors of fibroblast growth factor receptors (FGFR), phosphatidylinositol 3-kinase (PI3K) and Akt, i.e., SU5402, LY294002, and MK2206, respectively, bFGF failed to induce the *MAP2* mRNA expression and the neuron-like morphological change. bFGF induced Akt phosphorylation, but it was attenuated by the FGFR inhibitor SU5402 and the PI3K inhibitor LY294002. In canine BMSCs, expression of FGFR-1 and FGFR-2 was confirmed, but only FGFR-2 activation was detected by cross-linking and immunoprecipitation analysis. Small interfering RNA-mediated knockdown of FGFR-2 in canine BMSCs resulted in the attenuation of bFGF-induced Akt phosphorylation. These results suggest that the FGFR-2/PI3K/Akt signaling pathway is involved in the bFGF-induced neuronal differentiation of canine BMSCs.

## Introduction

Neuronal differentiation is a complex process coordinated by the down-regulation of pluripotent genes and concomitant up-regulation of neuron-specific lineage genes. Established cell culture models are frequently used to study *in vitro* neuronal differentiation. These models exhibit neuron-like morphology and express neuronal marker mRNAs and proteins in response to several neurotrophins, growth factors, and chemical compounds. Rat adrenal pheochromocytoma cells (PC12) differentiate into sympathetic neuron-like cells, which are characterized by neurite outgrowth, electrical excitability, and expression of neuronal markers in response to nerve growth factor (NGF) [1–3]. Mouse neuroblastoma cells (Neuro-2A) exhibited neuron-like morphology in the presence of the cannabinoid receptor agonist HU-210 [4, 5]. In human neuroblastoma cell lines (SK-N-SH, BE(2)-C, and NB1643), all-trans retinoic acid induced neurite outgrowth and expression of neuronal markers [6, 7]. Recently, adult tissue stem cells have been reported to possess neuronal differentiation potency, and considered as a useful tool for neuronal differentiation research [8, 9].

Bone marrow stromal cells (BMSCs) are fibroblastic adherent cells isolated from the bone marrow in adult human and animals such as mouse and dogs. Similar to other stem cell types, BMSCs have a high capacity for self-renewal, and are capable of differentiating into mesodermal cells, including adipocytes, chondrocytes, osteocytes, and ectodermal cells such as neurons and glial cells [10–14]. Cells undergo the following three steps in their differentiation into mature neurons: commitment of an ectodermal lineage, specialization of the neuronal lineage, and maturation of neuron characteristics. Previous studies using several animal models found that *in vitro* neuronal differentiation follows the processes of *in vivo* neuronal development, which are related to the surrounding microenvironments. Therefore, the arrangement of a proper microenvironment for the neuronal development process is essential to induce BMSCs into neurons. Treatment of rat BMSCs with chemical compounds such as β-mercaptoethanol and butylated hydroxyanisole resulted in the expression of neuron markers and neuron-like morphological changes [15]. Similarly, mice BMSCs treated with β-mercaptoethanol and brain-derived neurotrophic factor (BDNF) expressed neuron marker mRNAs, exhibited neuron-like morphologies, and voltage-dependent inward currents [16]. In human BMSCs, β-mercaptoethanol, butylated hydroxyanisole, and retinoic acid induced the expression of neuron marker mRNAs and proteins, but failed to induce the exhibition of voltage-dependent Na^+^ current [17]. On the other hand, human BMSCs were reported to express neuron marker mRNAs and proteins and exhibit functional α-amino-3-hydroxy-5-methyl-4-isoxazolepropionic acid (AMPA) receptors using cAMP, 3-isobutyl-1-methylxanthine, NGF and insulin [18].

Basic fibroblast growth factor (bFGF) functions as a differentiation factor as well as a neurotrophic factor in the central nervous system, where it is highly expressed. It supports cell survival, growth, and differentiation. [19–21]. bFGF contributes to the specification of the neuronal lineage in the *in vitro* neuronal differentiation process along with other extracellular molecules. In mouse BMSCs treated with bFGF, neuron-specific proteins, functional dopamine receptors, and voltage-dependent channels were expressed, and neuron-like K^+^ outward currents were detected [22, 23]. In human BMSCs, bFGF contributed to differentiation of BMSCs into functional neuron-like cells; these BMSCs expressed neuron-specific mRNAs and proteins as well as exerted voltage-responsive and dopamine-secreting neuron-like functions [24–27]. Moreover, we have previously reported that canine BMSCs treated with bFGF alone expressed neuron-specific mRNAs (microtubule associated protein-2 [*MAP2*], neurofilament light chain, and neuron-specific enolase) and proteins (neurofilament light chain and neuron-specific enolase), and exhibited neuron-like morphology. In the bFGF-treated canine BMSCs, KCl and L-glutamate evoked a sharp rise in intracellular Ca^2+^ concentrations, suggesting that bFGF induced differentiation of canine BMSCs into voltage- and glutamate-responsive neuron-like cells [28].

bFGF activates several signaling pathways such as MAPK/ERK kinase (MEK)/Extracellular signal-regulated kinase (ERK) pathway. In mouse BMSCs, bFGF induces neuronal differentiation via the MEK/ERK pathway [23]; therefore, activation of this pathway is considered crucial in bFGF-induced neuronal differentiation of mouse BMSCs. bFGF also activates the phosphoinositide 3-kinase (PI3K)/Akt pathway. Furthermore, bFGF mediates cell survival via the PI3K/Akt pathway in mouse models of neuronal differentiation, embryonic carcinoma cell lines (P19 cells), embryonic stem cells, and primary neural stem cells [29]. In PC12 cells, bFGF suppressed endoplasmic reticulum stress-induced apoptosis via the PI3K/Akt pathway [30, 31]. In this study, we examined the role of activation of PI3K/Akt pathway in bFGF-induced neuronal differentiation of canine BMSCs.

## Materials and Methods

### Isolation and Culture of Canine BMSCs

This study was approved by the Nihon University Animal Care and Use Committee (AP12B015). Three healthy beagles (male, 3 years old) were purchased from Japan SLC Inc., and bred and maintained in cages (height: 137 cm; width: 80 cm; length: 86 cm). The experimental food TC-2 (250 g/head; Oriental Yeast Co. Ltd.) was provided to all study animals once a day. The dogs were exercised using some toys inside (once a day) and outside (once a month) of the animal breeding facility. The physical conditions of the facility were monitored once a day. To avoid infection, the dogs were housed distantly from each other. All efforts were made to improve animal welfare and minimize discomfort. The dogs were premedicated with an intravenous injection of midazolam hydrochloride (0.2 mg/kg; Astellas Pharma Inc., Tokyo, Japan) and butorphanol tartrate (0.2 mg/kg; Meiji Seika Pharma Co. Ltd., Tokyo, Japan). Anesthesia was induced intravenously with propofol (4.0 mg/kg; Intervet K.K, Osaka, Japan) and maintained with 2.0% isoflurane (Intervet K.K.) and 100% oxygen was provided in an endotracheal tube. To minimize potential pain and infection, butorphanol tartrate (0.2 mg/kg) and cefazolin sodium hydrate (20 mg/kg, Astellas Pharma Inc.) were administered intravenously before awakening. Canine BMSCs were isolated by a previously described method [28, 32–34]. Briefly, canine bone marrow was aspirated from the humerus and mononuclear cells were separated by density-gradient centrifugation using Histopaque-1077 (Sigma-Aldrich Inc., St. Louis, MO). Thereafter, the mononuclear cells were then transferred to a 75-cm^2^ plastic culture flask (Thermo Fisher Scientific, Inc., St. Waltham, MA) and static-cultured in an incubator at 5% CO_2_ and 37°C using α-modified Eagle minimum essential medium (Life Technologies Co., Carlsbad, CA) supplemented with 10% fetal bovine serum (Life Technologies Co.). On the fourth day of culture, nonadherent cells were removed when the culture medium was replaced, thus isolating canine BMSCs. The cells were harvested using 0.25% trypsin-ethylenediaminetetraacetic acid (trypsin-EDTA; Life Technologies Co.) once they reached approximately 90% confluence. Then, the collected cells were seeded at a density of 14,000 cells/cm^2^. The second-passage canine BMSCs were used for all subsequent experiments.

### Neuronal Induction Using bFGF

Canine BMSCs were placed in a 25-cm^2^ plastic culture flask (Thermo Fisher Scientific, Inc.) at a density of 4,000 cells/cm^2^. bFGF-induced neuronal differentiation was performed as described previously [28]. In brief, the medium was changed to Neurobasal-A medium (Life Technologies Co.) supplemented with 2% B-27 supplement (Life Technologies Co.) and 100 ng/ml recombinant human bFGF (Immunostep, Salamanca, Spain) at 24 h of passage.

### Inhibitor Treatment

Canine BMSCs were placed in a 25-cm^2^ plastic culture flask at a density of 4,000 cells/cm^2^. The cells were pretreated with Neurobasal-A medium with 2% B-27 supplement containing the fibroblast growth factor receptor (FGFR) inhibitor SU5402 (20 μM; Sigma-Aldrich Inc.), the PI3K inhibitor LY294002 (50 μM; Cell Signaling Technology Japan K.K., Tokyo, Japan), the Akt inhibitor MK2206 (1 μM; Selleck Chemicals Llc., Houston, TX), the PLC inhibitor U73122 (8 μM; Sigma-Aldrich Inc.), or the MEK/ERK inhibitor U0126 (20 μM; Sigma-Aldrich Inc.) for 1 h, following a slightly modified version of previously reported methods [23]. Next, cells were treated with bFGF as described above. After 3 days of bFGF treatment, total RNA was extracted from each sample, which was then subjected to real-time RT-PCR to evaluate mRNA expression of *MAP2*.

### Reverse Transcription-Polymerase Chain Reaction (RT-PCR)

Total RNA was extracted from canine BMSCs using TRIzol^®^ reagent (Life Technologies Co.) according to the manufacturer’s instructions. Total RNA concentration was measured spectrophotometrically by reading absorbance at 260/280 nm. First-strand cDNA synthesis was carried out using 500 ng of total RNA using the PrimeScript RT Master Mix (TaKaRa Bio Inc., Shiga, Japan). PCRs were performed using 2 μl of first-strand cDNA in 10 μl total reaction volume, with primers specific for canine FGF receptor (FGFR)-1, FGFR-2, FGFR-3 and FGFR-4 (Table 1) and Ex Taq (TaKaRa Bio Inc.). PCRs were conducted using iCycler (Bio-Rad, Hercules, CA). The thermal cycler was programmed for initial denaturation at 94°C for 2 min, followed by 25 cycles of denaturation at 94°C for 30 sec, primer annealing at 55°C for 30 sec, and primer extension at 72°C for 30 sec. The PCR products were separated using 2% agarose gel electrophoresis, followed by ethidium bromide staining and visualization under UV light. mRNA expression levels in each sample were normalized to that of TATA box-binding protein [*TBP*].

**Table 1:** Primers used for RT-PCR.

Gene Name	GenBank ID	Primer sequences
*FGFR-1*	XM_003639562.1	F: 5′-ACCAAAGTGGCCGTGAAGATG-3′
		R: 5′-CAGCAGGTTGATGATGTTCTTGTG-3′
*FGFR-2*	NM_001003336.1	F: 5′-TCGAGGGTGGACCTTAGTTGAGA-3′
		R: 5′-TCAGTGTTTCAATTTCGACGATGAC-3′
*FGFR-3*	XM_545926.3	F: 5′-CTGGTGTCTGAGATGGAGATGATGA-3′
		R: 5′-GCAGGTATTCCCGCAGGTTG-3′
*FGFR-4*	XM_003434496.1	F: 5′-CAGAGGCCTTTGGCATGGA-3′
		R: 5′-TTATGTCGGCCGATCAGCTTC-3′
*TBP*	XM_863452	F: 5′-ACTGTTGGTGGGTCAGCACAAG-3′
		R: 5′-ATGGTGTGTACGGGAGCCAAG-3′

### Real-Time RT-PCR

Real-time RT-PCRs were performed as previously reported [28, 34]. Total RNAs were extracted from canine BMSCs using TRIzol^®^ reagent (Life Technologies Co.) according to the manufacturer’s instructions. First-strand cDNA synthesis was performed using 500 ng of total RNA, using PrimeScript^®^ RT Master Mix (TaKaRa Bio Inc.). Real-time RT-PCRs were performed with 2 μl of the first-strand cDNA in 25 μl total reaction volume, with canine-specific primers for the neuron marker *MAP2* (Table 2) and SYBR^®^ Premix Ex Taq™ II (TaKaRa Bio Inc.). The real-time RT-PCRs of no template controls were performed with 2 μl of RNase- and DNA-free water. In addition, real-time PCRs of no-reverse transcription controls were performed using 2 μl of each RNA sample. The PCRs were conducted using Thermal Cycler Dice^®^ Real Time System II (TaKaRa Bio Inc.). The PCR reactions consisted of 1 cycle of denaturing at 95°C for 30 sec, followed by 40 cycles of denaturing at 95°C for 5 sec and annealing and extension at 60°C for 30 sec. The specificity of each primer was previously verified using dissociation curve analysis and direct sequencing of each PCR product [34]. The results were analyzed by means of the second derivative method and the comparative cycle threshold method using TP900 DiceRealTime v4.02B (TaKaRa Bio Inc.). Amplification of β-glucuronidase (*GUSB*) from the same amount of cDNA was used as an endogenous control.

**Table 2:** Primers used for Real-time RT-PCR.

Gene Name	GenBank ID	Primer sequences
*MAP2*	XM_845165.1	F: 5′-AAGCATCAACCTGCTCGAATCC-3′
		R: 5′-GCTTAGCGAGTGCAGCAGTGAC-3′
*GUSB*	NM_001003191.1	F: 5′-ACATCGACGACATCACCGTCA-3′
		R: 5′-GGAAGTGTTCACTGCCCTGGA-3′

### Cross-Linking and Immunoprecipitation

Cross-linking and immunoprecipitation (CLIP) was performed as described previously [23], with some modifications. After incubation with bFGF (100 ng/ml) for 2 min, canine BMSCs were washed twice with cold PBS and cross-linked with 1 mM dithiobis(sulfosuccinimidyl propionate) disodium salt (DTSSP) at 4°C for 2 h. A fresh DTSSP stock solution was prepared in dimethyl sulfoxide. The reactions were terminated by the addition of glycine (final concentration of 100 mM) and incubation at room temperature for 15 min. The cells were washed twice with cold PBS and then lysed with lysis buffer (20 mM Tris-HCl, pH 7.5, 150 mM NaCl, 1 mM EDTA, 1 mM ethylene glycol tetraacetic acid, 1% Triton X-100, 2.5 mM Na_4_P_2_O_7_, 1 mM β-glycerophosphate, 1 mM Na_3_VO_4_, 1 mM phenylmethylsulfonyl fluoride, and complete mini EDTA-free protease inhibitor cocktail; from Roche, Mannheim, Germany) and centrifuged at 14,000 *g* for 20 min at 4°C. The proteins in the supernatant were quantified using DC™ protein assay reagent kit (Bio-Rad). For immunoprecipitation analysis, 500 μg of total cell lysates was precleared with protein A plus G Sepharose (GE Healthcare, Piscataway, NJ) before incubation with specific antibodies, followed by addition of protein A plus G Sepharose. Total cell lysate was incubated with 5 μg anti-bFGF antibody (Sigma-Aldrich Inc.) at 4°C for 18 h. The precipitated proteins were dissolved in sodium dodecyl sulfate (SDS) sample buffer before electrophoresis. Finally, the precipitated proteins were incubated in the presence or absence 200 mM dithiothreitol (DTT) and analyzed by western blotting with anti-FGFR-1 antibody (Abcam Plc., Cambridge, UK) or FGFR-2 antibody (Abcam Plc.).

### Western Blotting

Canine BMSCs before and after bFGF treatment were lysed with the lysis buffer containing 20 mM HEPES, 1 mM phenylmethylsulfonyl fluoride, 10 mM NaF, and complete mini EDTA-free protease inhibitor cocktail (Roche, Mannheim, Germany) at pH 7.4. Canine brain lysate obtained using the same lysis solution was used as a positive control for FGFR-1 and FGFR-2. Protein concentrations were normalized in accordance with Bradford’s method [35]. Extracted proteins were boiled at 95°C for 5 min in SDS-DTT buffer. Samples containing 30 μg of protein were loaded in each lane of a 7.5% polyacrylamide gel (Mini-PROTEAN TGX gel; Bio-Rad) and separated via electrophoresis. Thereafter, the separated proteins were transferred onto polyvinylidene difluoride membranes using Trans-Blot Turbo Transfer System (Bio-Rad). The membranes were blocked at room temperature for 50 min in Block Ace (DS Pharma Biomedical, Osaka, Japan), and then incubated with anti-FGFR-1 (1:250), FGFR-2 (1:1,000), phosphorylated Akt (p-Akt; Cell Signaling Technology Japan K.K., Tokyo, Japan, 1:1,000) or total Akt (t-Akt; Cell Signaling Technology Japan K.K., 1:1,000) antibody for 120 min at room temperature. After washing, the membranes were incubated with a horseradish peroxidase-conjugated anti-mouse, rabbit or goat IgG (GE Healthcare, 1:10,000) at room temperature for 90 min. Immunoreactivity was detected with ECL Western blotting Analysis System (GE Healthcare). The chemiluminescent signals of the membranes were measured, and densitometric analyses were performed using ImageQuant LAS 4000 mini (GE Healthcare).

### Small Interfering RNA (siRNA)

Canine BMSCs (4,000 cells/cm^2^) were placed in a 90-mm diameter plastic dish (Thermo Fisher Scientific, Inc.). The cells were transfected with 33 nM FGFR-2 siRNA (Sigma-Aldrich Inc.; Table 3) or 33 nM scramble siRNA (Sigma-Aldrich Inc.) as scramble control, combined with Lipofectamine 2000 (Life Technologies Co.) for 24 h, and then, bFGF treatment was performed as described above. After the treatment, total RNAs and proteins were extracted from each sample. Real-time RT-PCRs were performed to evaluate the mRNA expression of *FGFR-2*, and western blotting was performed to evaluate the phosphorylation of Akt (as described above).

**Table 3:** siRNA sequence for canine FGFR-2.

Gene Name	GenBank ID	siRNA sequences
FGFR-2	NM_001003336.1	5ʹ-GAGAUAGCCAUUUACUGCATT-3ʹ.

### Statistical Analysis

Data are presented as mean ± standard error (SE). Statistical analyses were performed using StatMate IV (ATMS, Tokyo, Japan). Data were analyzed using two-way analysis of variance. Tukey’s test was used as post-hoc analysis, and *P*-values less than 0.05 were considered statistically significant.

## Results

### bFGF Induces Neuronal Differentiation of Canine BMSCs via FGFR

**Fig 1:**
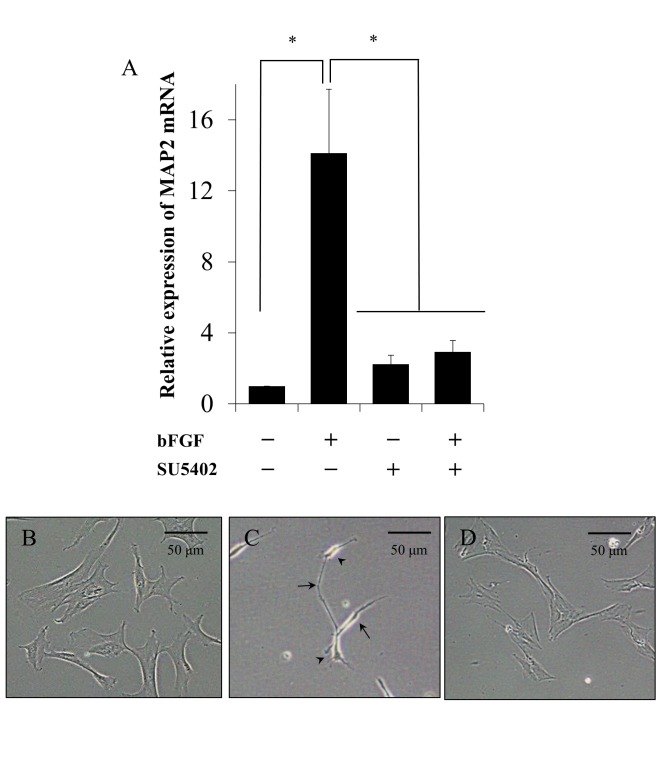
The FGFR inhibitor SU5402 attenuates bFGF-induced *MAP2* mRNA expression and neuron-like morphological change of canine BMSCs. A, After pretreatment with SU5402 (20 μM) for 1 h, canine BMSCs were incubated with bFGF (100 ng/ml) for 3 days. SU5402 completely suppressed bFGF-induced *MAP2* mRNA expression. Results are presented as means ± SE. n = 3. *P < 0.05. B, The morphology of untreated canine BMSCs was flattened and fibroblast-like. C, bFGF-treated cells exhibited neuron-like morphology, which was characterized by small cell bodies (arrowheads) and several long sharp processes like dendrites and axons (arrows). D, bFGF-induced morphological changes of the cells were inhibited in the presence of SU5402, which maintained a fibroblast-like shape.

We have previously reported that mRNA expression of the neuron marker *MAP2* and neuron-like morphology were observed in bFGF-induced neuronal cell differentiation of canine BMSCs by bFGF [28]. Therefore, to elucidate the signaling pathway involved in bFGF-induced neuronal differentiation of canine BMSCs, we first examined the effect of the FGFR inhibitor SU5402 (20 μM) on bFGF-induced *MAP2* mRNA expression. bFGF significantly induced *MAP2* mRNA expression in the absence of this inhibitor, but its effect was attenuated in the presence of the inhibitor (Fig 1A). Basal *MAP2* mRNA expression was unaffected by the inhibitor compared to untreated cells, and the expression of the housekeeping gene *GUSB* remained stable for 3 days.

Next, we checked the effect of the FGFR inhibitor on morphological changes of bFGF-treated cells. Untreated canine BMSCs exhibited a flattened and fibroblast-like morphology (Fig 1B). bFGF treatment resulted in canine BMSCs exhibiting neuron-like morphology, which was characterized by a small cell body and several long and sharp processes; this change was observed within 3 days of treatment (Fig 1C). However, the FGFR inhibitor inhibited the bFGF-induced neuron-like morphological changes (Fig 1D). No effect of these inhibitors on the viability of BMSCs was verified by trypan blue exclusion assay. The FGFR inhibitor was previously reported to attenuate the bFGF-induced neuron marker expression and morphological changes in mouse BMSCs [23]. In rat microglia, the FGFR inhibitor inhibited bFGF-promoted generation of MAP2-positive cells [36]. Therefore, our observations suggest that bFGF-induced neuronal differentiation in canine BMSCs is largely dependent on FGFR.

### Binding of bFGF with FGFR-2

**Fig 2:**
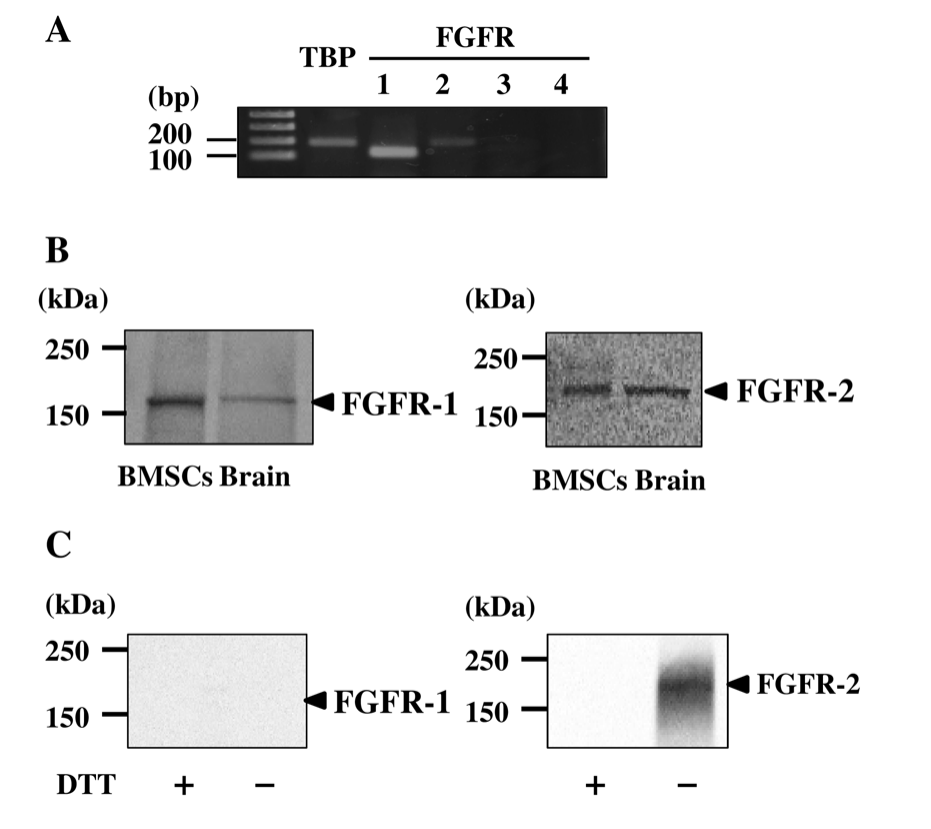
Expression of various FGFR subtypes and binding of bFGF to these subtypes in canine BMSCs. A, mRNA expression of four different subtypes of FGFR determined by RT-PCR using total RNA extracted from canine BMSCs. PCR products for *FGFR-1* and *FGFR-2* were detected to be 120 and 193 bp, respectively. *TBP* control was used as an internal standard for the PCR analysis. B, Detection of FGFR-1 and FGFR-2 proteins (arrowheads) by western blotting using anti-FGFR-1 and anti-FGFR-2 antibodies. Canine brain extracts served as positive controls. C, Canine BMSCs were incubated with bFGF (100 ng/ml) for 2 min and cross-linked with DTSSP (1 mM) at 4°C for 2 h. Whole-cell proteins were extracted and immunoprecipitated with anti-bFGF antibody. Finally, the precipitated bFGF/FGFR complex was treated in the presence or absence DTT (200 mM) and detected by western blotting with anti-FGFR-1 or anti-FGFR-2 antibody. FGFR-2 protein was clearly detected after CLIP in the absence of DTT as indicated by an arrowhead.

Four subtypes of FGFRs have been identified in mammals [37]; therefore, to determine which of these subtypes contributes to the specific neurogenic effects of bFGF in canine BMSCs, we examined the mRNA and protein expression of FGFR subtypes. mRNA expression of *FGFR-1* and *FGFR-2* was detectable, whereas that of *FGFR-3* and *FGFR-4* was undetectable by RT-PCR experiments using canine BMSCs (Fig 2A). Expression of FGFR-1 and FGFR-2 proteins in canine BMSCs was confirmed by western blotting (Fig 2B); these results suggested that FGFR-1 and FGFR-2 are the major subtypes of FGFR in canine BMSCs.

Because bFGF is known to show varying binding affinities for various FGFR subtypes [38], we further analyzed binding of bFGF with FGFR subtypes by CLIP experiments. After incubation with bFGF, FGFR-2 was clearly detectable by the CLIP experiments, whereas FGFR-1 was undetectable (Fig 2C). These results indicate that bFGF mainly bound to FGFR-2 on the surface of BMSCs.

### bFGF Induced Neuronal Differentiation Depends on the Activation of PI3K/Akt Pathway

**Fig 3:**
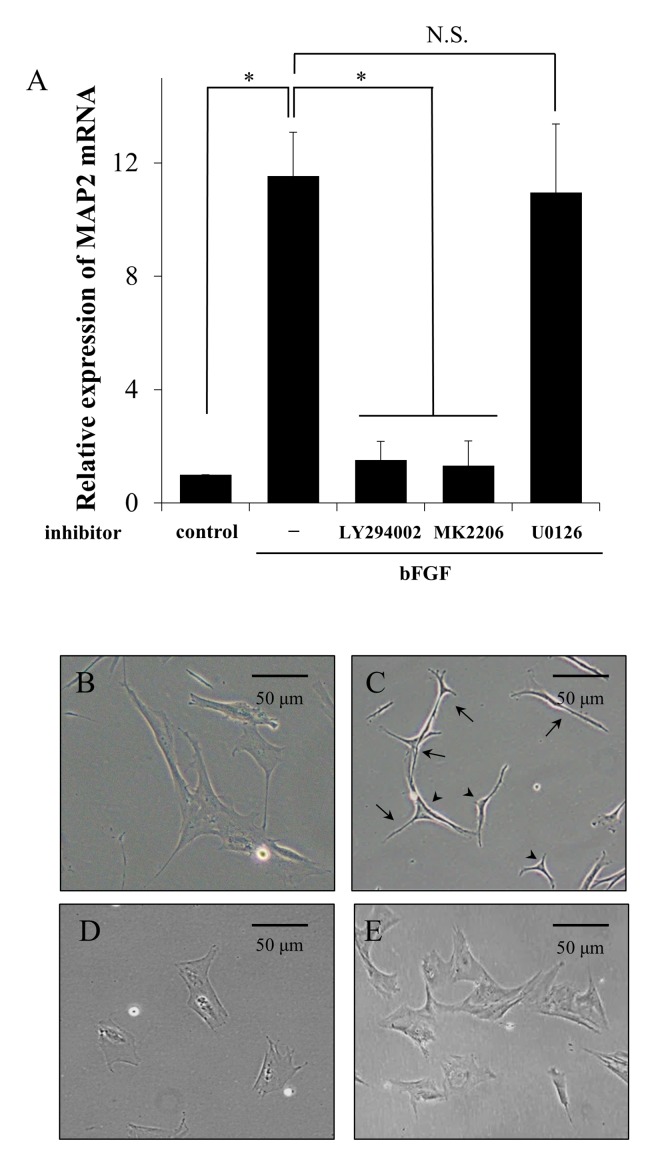
PI3K and Akt inhibitors, but not PLC and MEK/ERK inhibitors, attenuate bFGF-induced *MAP2* mRNA expression and neuron-like morphological changes in canine BMSCs. A, BMSCs pretreated with the PI3K inhibitor LY294002 (50 μM), the Akt inhibitor MK2206 (1 μM), the MEK/ERK inhibitor U0126 (20 μM), and the PLC inhibitor U73122 (8 μM) for 1 h were incubated with bFGF (100 ng/ml) for 3 days. bFGF-induced *MAP2* mRNA expression was completely inhibited in cells treated with LY294002 and MK2206, but not in cells treated with U0126 and U73122. Results are presented as means ± SE. n = 3. *P < 0.05. B, Untreated canine BMSCs exhibited fibroblast-like morphology, which had flattened cell body. C, In the presence of bFGF, the cells exhibited neuron-like morphology, characterized by small cell bodies (arrowheads) and several long sharp processes such as dendrites and axons (arrows). D-F, In the presence of the PI3K (D) and Akt inhibitors (E), bFGF-induced morphological changes of the cells were inhibited, which maintained fibroblast-like shape.

To investigate the downstream signaling pathway of FGFR in canine BMSCs, we examined effect of the PI3K inhibitor LY294002 (50 μM), the Akt inhibitor MK2206 (1 μM), and the MEK/ERK inhibitor U0126 (20 μM) on bFGF-induced *MAP2* mRNA expression. bFGF significantly induced *MAP2* mRNA expression in the absence of these inhibitors (Fig 3A). In the presence of the PI3K and Akt inhibitors, the bFGF-induced *MAP2* mRNA expression was attenuated (Fig 3A). However, the MEK/ERK inhibitor had no effect on the bFGF-induced *MAP2* mRNA expression (Fig 3A). The expression of the housekeeping gene *GUSB* remained stable for 3 days.

We next investigated the morphology of bFGF-treated cells in the presence of PI3K and Akt inhibitors. Canine BMSCs showed fibroblast-like shape in the absence of bFGF (Fig 3B). The bFGF-treated cells exhibited neuron-like morphology within 3 days of treatment (Fig 3C). In the presence of the PI3K and Akt inhibitors, bFGF failed to induce neuron-like morphological change (Fig 3D and 3E). Furthermore, no effect of these inhibitors on viability of the cells was verified by the trypan blue exclusion assay.

The MEK/ERK inhibitor U0126 is known to inhibit bFGF-induced neuronal differentiation of mouse BMSCs [23]. However, in the present study, unlike the PI3K and Akt inhibitors, the MEK/ERK inhibitor had no effect on the bFGF-induced neuronal differentiation of canine BMSCs. These results suggest that the signaling pathway of neuronal differentiation in canine BMSCs differs from that previously reported in a mouse model, which depends on the activation of the PI3K/Akt pathway.

### bFGF induces Akt phosphorylation through FGFR and PI3K

**Fig 4:**
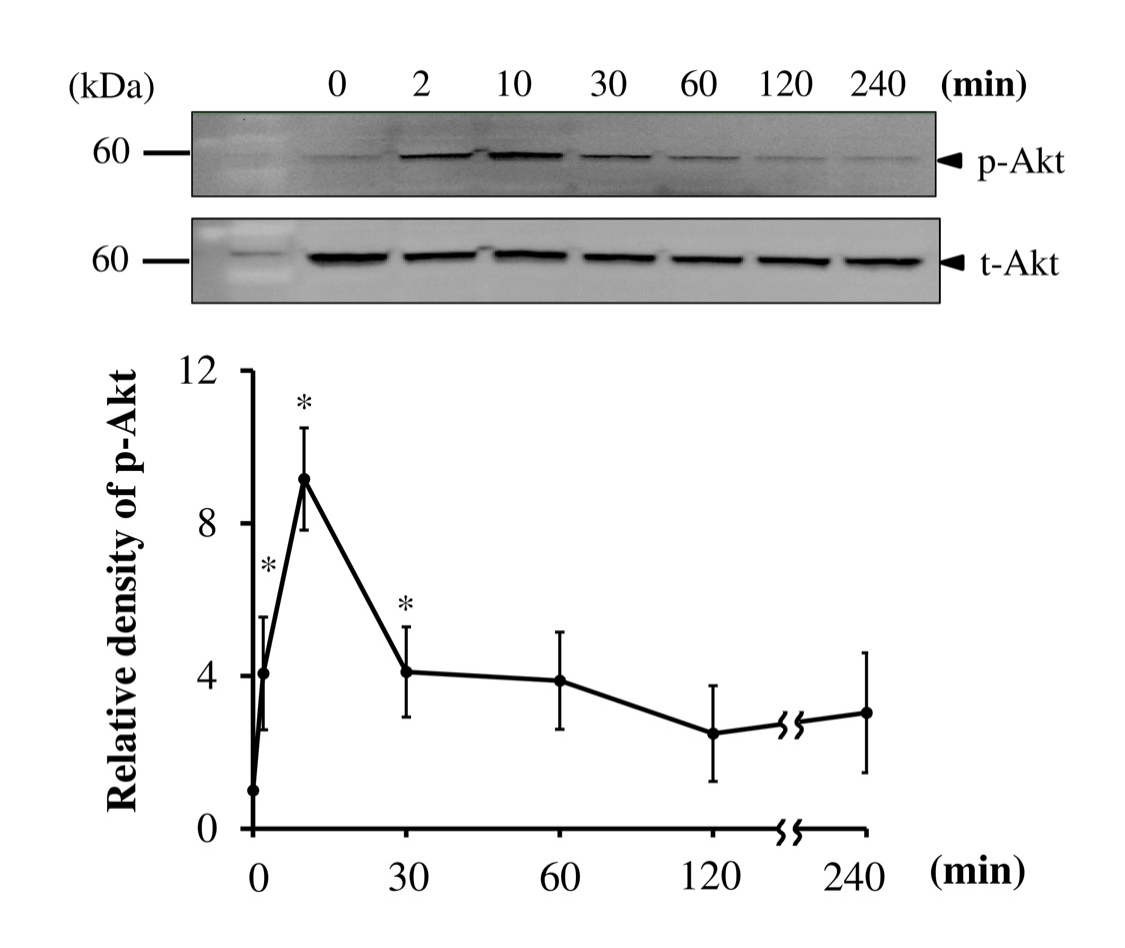
bFGF stimulates Akt phosphorylation in canine BMSCs. Western blotting for detection of phosphorylated Akt (p-Akt) in BMSCs treated with bFGF (100 ng/ml) for the indicated times (upper panel). Relative density of p-Akt compared with the results at 0 time (lower panel). Relative density of total Akt (t-Akt) compared with the results at 0 time (lower panel). bFGF stimulated the phosphorylation of Akt in a time-dependent manner. Results are presented as means ± SE. n = 3. *p < 0.05.

**Fig 5:**
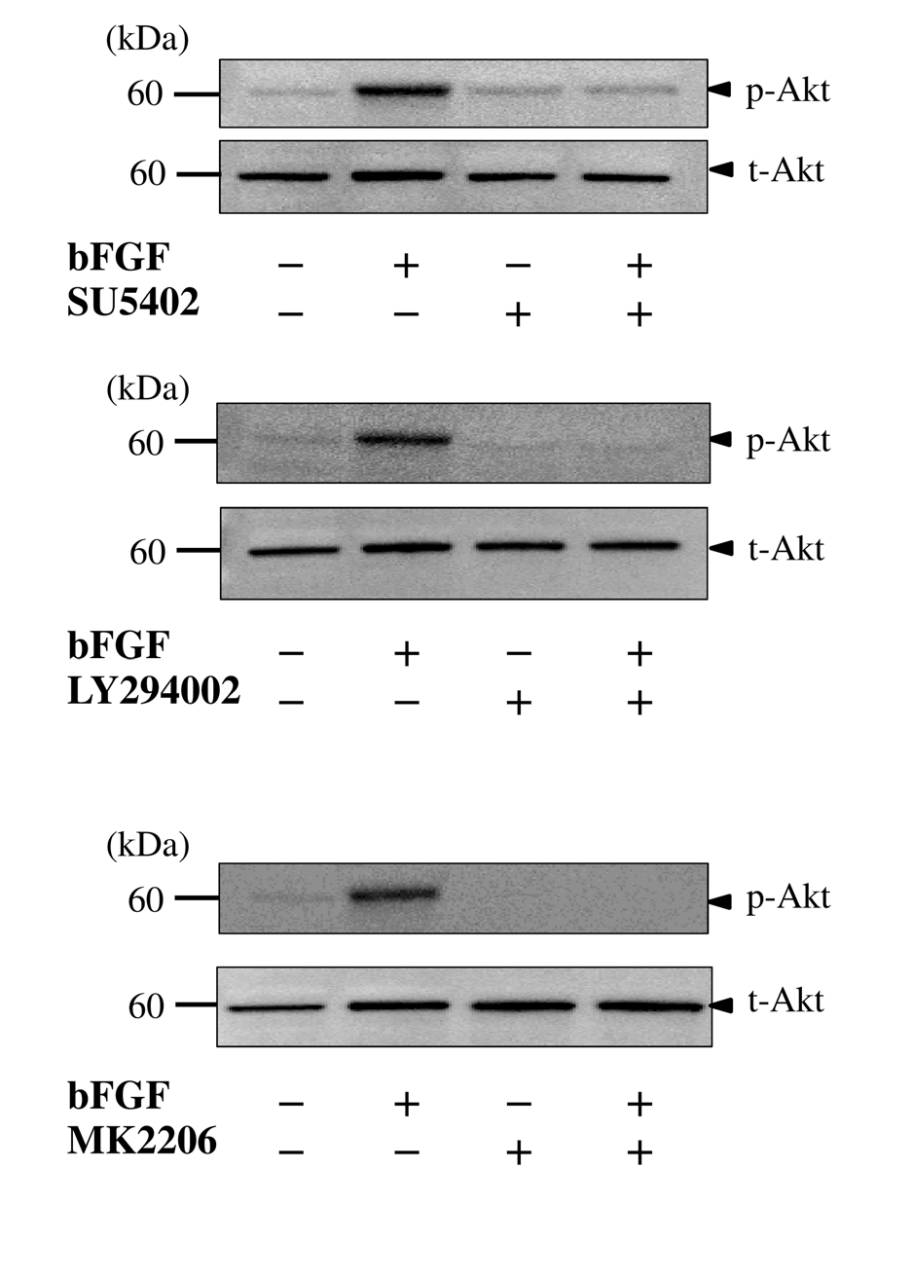
FGFR, PI3K, and Akt inhibitors attenuate bFGF-induced Akt phosphorylation. After pretreatment with the FGFR inhibitor SU5402 (25 μM), the PI3K inhibitor LY294002 (50 μM), and the Akt inhibitor MK2206 (1 μM) for 1 h, BMSCs were incubated with bFGF (100 ng/ml) for 10 min. Phosphorylation of Akt was examined by western blotting. The inhibitors of FGFR, PI3K, and Akt completely suppressed bFGF-induced Akt phosphorylation (arrowheads).

A serine/threonine kinase Akt is a downstream target of PI3K and is activated by phosphorylation within the carboxy-terminus at serine 473 [39]. To verify whether bFGF activates the PI3K/Akt pathway, we examined the activation of Akt using an anti-p-Akt antibody. Akt phosphorylation occurred after bFGF treatment in a time-dependent manner, which peaked at 10 min (Fig 4). To confirm whether the Akt phosphorylation is dependent on FGFR and PI3K, we examined the effects of the FGFR inhibitor SU5402, the PI3K inhibitor LY294002 and the Akt inhibitor MK2206 on bFGF-induced Akt phosphorylation. We found that all three inhibitors completely inhibited bFGF-induced phosphorylation of Akt (Fig 5). These observations strongly suggest that bFGF induces activation of Akt by its phosphorylation via FGFR and PI3K.

### FGFR-2 Contributes to bFGF-Induced Akt Activation

**Fig 6:**
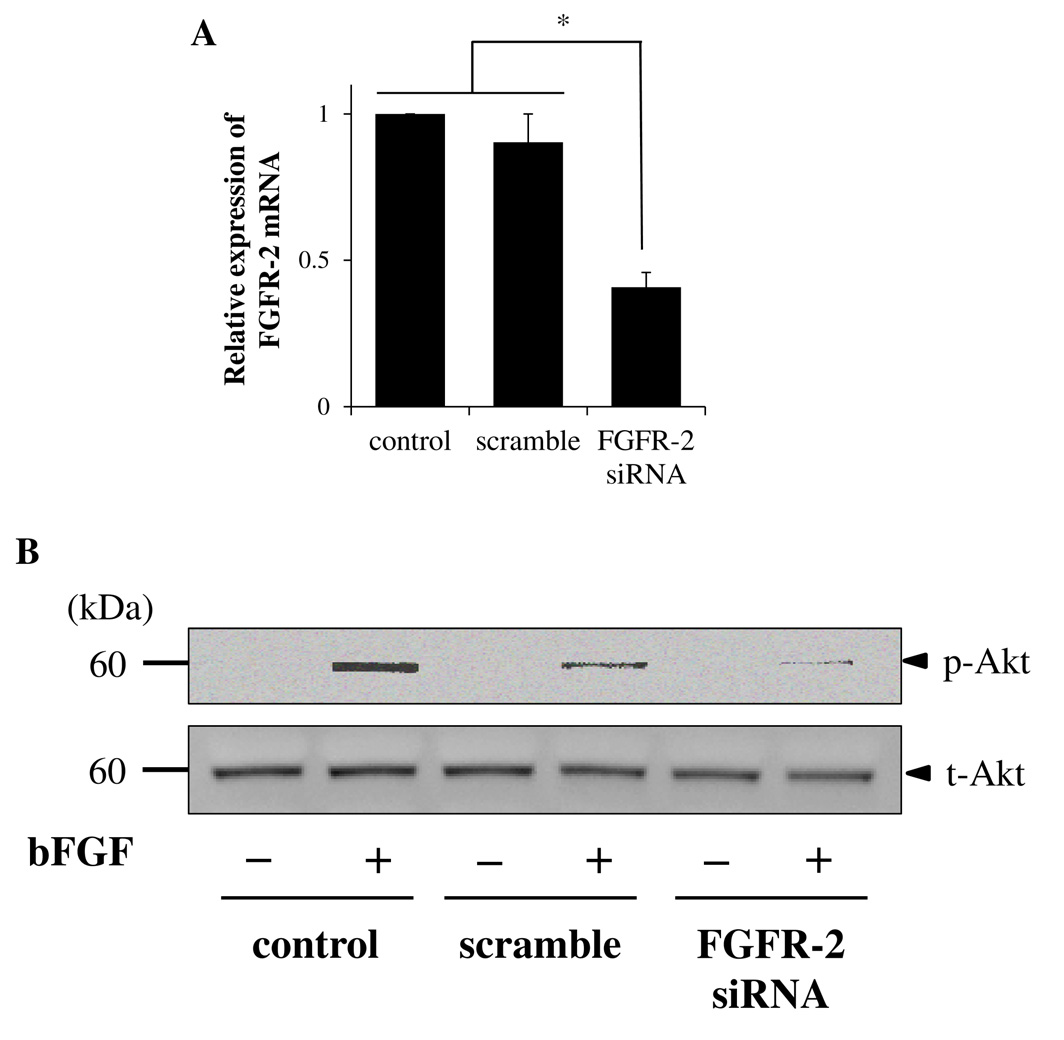
FGFR-2 siRNA inhibits the bFGF-induced neuronal differentiation signaling pathway. A, FGFR-2 siRNA transfection of canine BMSCs resulted in a significant decrease of *FGFR-2* mRNA expression but not control or scramble siRNA transfection. B, Western blotting for detection of bFGF-induced Akt phosphorylation in control, scramble siRNA-transfected, or FGFR-2 siRNA-transfected cells. FGFR-2 siRNA transfection clearly inhibited the bFGF-induced Akt phosphorylation compared with the control, but to a lesser extent scramble siRNA.

To elucidate whether FGFR-2 plays a central role in the activation of the PI3K/Akt pathway, we performed an FGFR-2 knockdown experiment using FGFR-2 siRNA transfection. As shown in Fig 6A, *FGFR-2* mRNA expression was significantly decreased by FGFR-2 siRNA transfection, but not by control and scramble siRNA transfection. Thereafter, we examined for Akt phosphorylation in control cells, scramble siRNA-transfected cells, and FGFR-2 siRNA-transfected cells. Akt phosphorylation was clearly inhibited by FGFR-2 siRNA transfection compared with the control, but to a lesser extent by scramble siRNA transfection (Fig 6B). These results strongly suggest that FGFR-2 activation contributes to activation of the PI3K/Akt signaling pathway, which attributes to bFGF-induced neuronal differentiation of canine BMSCs.

## Discussion

In this study, we demonstrated that FGFR-2 activation contributes to bFGF-induced neuronal differentiation through the activation of the PI3K/Akt signaling pathway in canine BMSCs.

In humans, rodents, and other species, such as zebrafish, amphibians, and chickens, four subtypes of FGFR, FGFR-1 through FGFR-4, have been identified [40–42]. In canine BMSCs, we previously demonstrated that bFGF predominantly bound to FGFR-2, which is thought to be mostly associated with neuronal development in mammals [40, 43]. An *FGFR-2* null mutation in mice causes early mortality, prior to the formation of a mature central nervous system [40, 43]. Mutations in *FGFR-2* lead to aberrant neuronal development or to neuronal diseases in humans, such as megalocephaly, midline disorders, hippocampal malformations, and ventricular wall alterations [44, 45]. Therefore, FGFR-2 is likely to be involved in the bFGF-induced neuronal differentiation of canine BMSCs.

bFGF has previously been reported to activate MEK/ERK pathways and consequently induce neuronal differentiation in rat pheochromocytoma cells (PC12), mouse or human neuroblastoma cell lines (Neuro2A, SK-N-SH and BE(2)-C), and embryonic stem cells [2, 3, 5, 7, 46]. In mouse BMSCs, a MEK/ERK inhibitor attenuated bFGF-induced neuronal differentiation, but a PI3K inhibitor failed to induce the bFGF effect [23]. In mouse cells, bFGF-induced phosphorylation of MEK and ERK was observed, but Akt phosphorylation was not [23]. Therefore, MEK/ERK pathways are considered to play a central role in bFGF-induced neuronal differentiation. In contrast, in our study, PI3K and Akt inhibitors clearly attenuated bFGF-induced neuronal marker expression and morphological change in canine BMSCs, but the MEK/ERK inhibitor did not show this effect. Furthermore, we confirmed that bFGF induces the phosphorylation of Akt through FGFR and PI3K. These results strongly suggest that the PI3K/Akt pathway contributes to the bFGF-induced neuronal differentiation of canine BMSCs. The PI3K/Akt pathway has been shown to play important roles in the regulation of cytoskeletal rearrangement, membrane expansion, transcription, and translation [47]. PI3K plays a fundamental role in regulating neuronal generation through the activation of Akt [48, 49]. PI3K phosphorylates the membrane phospholipid phosphatidylinositol 4,5-phosphate (PIP_2_), converting it to phosphatidylinositol 3,4,5-trisphosphate (PIP_3_). PIP_2_ and PIP_3_ in turn cause the activation of Akt [47, 50]. The accumulation of PIP_3_ promotes the translocation of Akt to the plasma membrane, where Akt binds to PIP_3_ via its PH domain, allowing phosphorylation of the threonine-308 residue on Akt by phosphoinositide-dependent kinase 1. The maximal activation of Akt requires the additional phosphorylation of serine-473 in the regulatory domain, although protein kinases involved in this phosphorylation are still obscure [51–53]. The constitutively active form of Akt initiates neurite elongation only in early differentiation stages [54]. The overexpression of constitutively active Akt induces neurite outgrowth and the expression of neuronal markers [7, 39]. Activated Akt promotes neuronal differentiation in neural stem cells [49, 55, 56]. It is therefore conceivable that PI3K/Akt pathway contributes to neuronal differentiation in canine BMSCs.

To investigate whether FGFR-2 activates the PI3K/Akt signaling pathway, we performed a FGFR-2 knockdown experiment using the transfection of FGFR-2 siRNA. We showed that knocking down FGFR-2 clearly inhibited bFGF-induced Akt phosphorylation. These observations strongly suggest that FGFR-2 has a crucial function, via the PI3K/Akt pathway, in the neuronal differentiation of canine BMSCs. On the other hand, bFGF has been reported to bind to FGFR-1 and play a central role in the bFGF-induced neuronal differentiation of mouse BMSCs via the MEK/ERK signaling pathway [23]. Therefore, FGFR expression patterns and the binding affinity of bFGF to FGFRs appear to be attributable to the selection of signaling pathways.

The downstream target of the FGFR-2/PI3K/Akt pathway in bFGF-induced neuronal differentiation of canine BMSCs is still obscure. In mouse BMSCs, bFGF-induced activation of the MEK/ERK pathway has been reported to activate transcription factor AP-1, which is subsequently involved in neuronal differentiation [23]. We therefore examined the effect of bFGF on AP-1 activation in canine BMSCs. However, we observed that bFGF had no effect on the activation of AP-1 (data not shown), suggesting that transcription factors distinct from AP-1 are a downstream target of the FGFR-2/PI3K/Akt pathway. Akt phosphorylates and inhibits glycogen synthase kinase-3β (GSK-3β); this inhibition leads to the activation of a transcriptional co-activator β-catenin, which consequently induces neuronal differentiation. In human neural stem cells, the GSK-3β/β-catenin pathway is involved in motor neuron differentiation [57–60]. This pathway also mediates neuronal differentiation in human BMSCs [61, 62]. The mammalian target of rapamycin (mTOR) is also in a pathway downstream of Akt. In human neural progenitor cells, mTOR activates P70S6K, and consequently induces neuronal differentiation, although P70S6K targets involved in this process have not been investigated in detail [63]. In canine adipose-derived stem cells, mTOR/p70S6K activation stimulated by PI3K/Akt participates in neuronal differentiation [64]. Therefore, we hypothesize that such factors are may mediate the neuronal differentiation of canine BMSCs. Further study of this possibility is underway in our laboratory.

Currently, only bFGF has been reported to induce the differentiation of human BMSCs into dopaminergic neurons. However, the downstream signaling of bFGF in this process is obscure [27]. In human BMSCs, multiple signaling pathways, including MEK/ERK and PI3K/Akt, have been reported to regulate neuronal differentiation [65]. In mice, bFGF has been shown to induce the activation of the FGFR-1/MEK/ERK pathway but not the PI3K/Akt pathway in BMSCs [23]. In the present study, we demonstrated the activation of FGFR-2/PI3K/Akt signaling in bFGF-treated canine BMSCs. The previous reports and our observations suggest that differences in signal transduction mechanisms in neuronal differentiation are probably dependent on species, intrinsic cellular processes, and the extracellular microenvironment. Therefore, we hypothesize that canine models could partially mimic aspects of human neuronal differentiation, making canine BMSCs useful as a model to understand the mechanisms of neuronal differentiation and its regeneration in humans.

In human BMSCs, not only bFGF but also neurotrophins (NTs), glial cell line-derived neurotrophic factor (GDNF) and Wnt have been reported to induce the differentiation into neurons [24, 62, 67–70]. NTs including nerve growth factor, brain-derived neurotrophic factor, neurotrophin-3, neurotrophin-4/5, promote the survival and growth of developing neurons, and axon regrowth in SCI, maintain the function of mature neurons, and prevent neuron death. Neurotrophin-3 induced the differentiation of human BMSCs into GABAergic neuron, which exhibited spontaneous post-synaptic currents, but none of them exerted action potentials [68–70]. Nerve growth factor and brain-derived neurotrophic factor induced the differentiation of human BMSCs into cholinergic neuron-like cells in the presence of Wnt 7a [62]. GDNF, involving with the generation and development of midbrain dopaminergic neurons, induced the expression of dopaminergic neuron markers in human BMSCs [24, 67]. Contribution of epidermal growth factor, Sonic hedgehog, FGF-8, and all-trans retinoic acid to the differentiation of human BMSCs into dopaminergic neuron-like cells in the presence or absence of bFGF has also been reported [27, 66]. These observations imply that such exogenous factors affect intracellular signaling pathways, which results in the differentiation of BMSCs into specific neurotransmitter-responsive or neurotransmitter-supplying neurons. It may facilitate the treatment of neuronal diseases to provide such specific neurons derived from BMSCs by activating signaling pathways. Therefore, the results of this study could further the future use of BMSCs in cellular replacement therapy.

## Conclusions

In conclusion, we demonstrated that the FGFR-2/PI3K/Akt pathway contributes to bFGF-induced neuronal differentiation of canine BMSCs. Our results provide new insights into the bFGF-induced neuronal differentiation mechanism, and may enable the development of new cell-based treatments for neuronal diseases.

## References

[1] Greene LA, Tischler AS, Establishment of a noradrenergic clonal line of rat adrenal pheochromocytoma cells which respond to nerve growth factor. Proc Natl Acad Sci U S A. 1976; 73: 2424–2428. 1065897 10.1073/pnas.73.7.2424 PMC430592

[2] Rydel RE, Greene LA, Acidic and basic fibroblast growth factors promote stable neurite outgrowth and neuronal differentiation in cultures of PC12 cells. J Neurosci 1987; 7: 3639–3653. 3316527 10.1523/JNEUROSCI.07-11-03639.1987 PMC6569034

[3] Mullenbrock S, Shah J, Cooper GM, Global expression analysis identified a preferentially nerve growth factor-induced transcriptional program regulated by sustained mitogen-activated protein kinase/extracellular signal-regulated kinase (ERK) and AP-1 protein activation during PC12 cell differentiation. J Biol Chem. 2011; 286: 45131–45145. 10.1074/jbc.M111.274076 22065583 PMC3248011

[4] He JC, Gomes I, Nguyen T, Jayaram G, Ram PT, Devi LA, et al The G α_o/i_-coupled cannabinoid receptor-mediated neurite outgrowth involves Rap regulation of Src and Stat3, J. Biol. Chem. 2005; 280: 33426–33434. 16046413 10.1074/jbc.M502812200

[5] Ma'ayan A, Jenkins SL, Barash A, Iyengar R, Neuro2A differentiation by Gα_o/i_ pathway. Sci Signal. 2009; 2: cm1, 10.1126/scisignal.254cm1 19155528 PMC2649824

[6] Middlemas DS, Kihl BK, Zhou J, Zhu X, Brain-derived neurotrophic factor promotes survival and chemoprotection of human neuroblastoma cells. J Biol Chem. 1999; 274: 16451–16460. 10347207 10.1074/jbc.274.23.16451

[7] Qiao J, Paul P, Lee S, Qiao L, Josifi E, Tiao JR, et al PI3K/AKT and ERK regulate retinoic acid-induced neuroblastoma cellular differentiation. Biochem Biophys Res Commun. 2012; 424: 421–426. 10.1016/j.bbrc.2012.06.125 22766505 PMC3668681

[8] Lobo MV, Alonso FJ, Redondo C, López-Toledano MA, Caso E, Herranz AS, et al Cellular characterization of epidermal growth factor-expanded free-floating neurospheres. J Histochem Cytochem. 2003; 51: 89–103. 12502758 10.1177/002215540305100111

[9] Chu MS, Chang CF, Yang CC, Bau YC, Ho LL, Hung SC, Signalling pathway in the induction of neurite outgrowth in human mesenchymal stem cells. Cell. Signal. 2006; 18: 519–530. 16098715 10.1016/j.cellsig.2005.05.018

[10] Prockop DJ, Marrow stromal cells as stem cells for nonhematopoietic tissues. Science. 1997; 276: 71–74. 9082988 10.1126/science.276.5309.71

[11] Poulsom R, Alison MR, Forbes SJ, Wright NA, Adult stem cell plasticity. J Pathol 2002; 197: 441–456. 12115861 10.1002/path.1176

[12] Vats A, Bielby RC, Tolley NS, Nerem R, Polak JM, Stem cells. Lancet. 2005; 366: 592–602. 16099296 10.1016/S0140-6736(05)66879-1

[13] Bahat-Stroomza M, Barhum Y, Levy YS, Karpov O, Bulvik S, Melamed E, et al Induction of adult human bone marrow mesenchymal stromal cells into functional astrocyte-like cells: potential for restorative treatment in Parkinson's disease. J Mol Neurosci. 2009; 39: 199–210. 10.1007/s12031-008-9166-3 19127447

[14] Kaka GR, Tiraihi T, Delshad A, Arabkheradmand J, Kazemi H, In vitro differentiation of bone marrow stromal cells into oligodendrocyte-like cells using triiodothyronine as inducer. Int J Neurosci. 2012; 122: 237–247. 10.3109/00207454.2011.642037 22115181

[15] Woodbury D, Schwarz EJ, Prockop DJ, Black IB, Adult rat and human bone marrow stromal cells differentiate into neurons. J Neurosci Res. 2000; 61: 364–370. 10931522 10.1002/1097-4547(20000815)61:4<364::AID-JNR2>3.0.CO;2-C

[16] Egusa H, Schweizer FE, Wang CC, Matsuka Y, Nishimura I, Neuronal differentiation of bone marrow-derived stromal stem cells involves suppression of discordant phenotypes through gene silencing. J Biol Chem. 2005; 280: 23691–23697. 15855172 10.1074/jbc.M413796200

[17] Mareschi K, Novara M, Rustichelli D, Ferrero I, Guido D, Carbone E, et al Neural differentiation of human mesenchymal stem cells: Evidence for expression of neural markers and eag K^+^ channel types. Exp Hematol. 2006; 34: 1563–1572. 17046576 10.1016/j.exphem.2006.06.020

[18] Tondreau T, Dejeneffe M, Meuleman N, Stamatopoulos B, Delforge A, Martiat P, et al Gene expression pattern of functional neuronal cells derived from human bone marrow mesenchymal stromal cells. BMC Genomics. 2008; 9: 166, 10.1186/1471-2164-9-166 18405367 PMC2358905

[19] Weiss S, Dunne C, Hewson J, Wohl C, Wheatley M, Peterson AC, et al Multipotent CNS stem cells are present in the adult mammalian spinal cord and ventricular neuroaxis. J Neurosci. 1996; 16: 7599–7609. 8922416 10.1523/JNEUROSCI.16-23-07599.1996 PMC6579089

[20] Frebel K, Wiese S, Signalling molecules essential for neuronal survival and differentiation. Biochem Soc Trans. 2006; 34: 1287–1290. 17073803 10.1042/BST0341287

[21] Sun D, Bullock MR, McGinn MJ, Zhou Z, Altememi N, Hagood S, et al Basic fibroblast growth factor-enhanced neurogenesis contributes to cognitive recovery in rats following traumatic brain injury. Exp Neurol. 2009; 216: 56–65. 10.1016/j.expneurol.2008.11.011 19100261 PMC2707259

[22] Tropel P, Platet N, Platel JC, Noël D, Albrieux M, Benabid AL, et al Functional neuronal differentiation of bone marrow-derived mesenchymal stem cells. Stem Cells. 2006; 24: 2868–2876. 16902198 10.1634/stemcells.2005-0636

[23] Yang H, Xia Y, Lu SQ, Soong TW, Feng ZW, Basic fibroblast growth factor-induced neuronal differentiation of mouse bone marrow stromal cells requires FGFR-1, MAPK/ERK, and transcription factor AP-1. J Biol Chem. 2008; 283: 5287–5295. 10.1074/jbc.M706917200 18171671

[24] Dezawa M, Kanno H, Hoshino M, Cho H, Matsumoto N, Itokazu Y, et al Specific induction of neuronal cells from bone marrow stromal cells and application for autologous transplantation. J Clin Invest. 2004; 113: 1701–1710. 15199405 10.1172/JCI20935 PMC420509

[25] Neuhuber B, Gallo G, Howard L, Kostura L, Mackay A, Fischer I, Reevaluation of in vitro differentiation protocols for bone marrow stromal cells: disruption of actin cytoskeleton induces rapid morphological changes and mimics neuronal phenotype. J Neurosci Res. 2004; 77: 192–204. 15211586 10.1002/jnr.20147

[26] Trzaska KA, King CC, Li KY, Kuzhikandathil EV, Nowycky MC, Ye JH, et al Brain-derived neurotrophic factor facilitates maturation of mesenchymal stem cell-derived dopamine progenitors to functional neurons. J Neurochem. 2009; 110: 1058–1069. 10.1111/j.1471-4159.2009.06201.x 19493166

[27] Nandy SB, Mohanty S, Singh M, Behari M, Airan B, Fibroblast Growth Factor-2 alone as an efficient inducer for differentiation of human bone marrow mesenchymal stem cells into dopaminergic neurons. J Biomed Sci. 2014; 21: 83, 10.1186/s12929-014-0083-1 25248378 PMC4190371

[28] Nakano R, Edamura K, Nakayama T, Teshima K, Asano K, Narita T, et al Differentiation of canine bone marrow stromal cells into voltage- and glutamate-responsive neuron-like cells by basic fibroblast growth factor. J Vet Med Sci. 2014; 77: 27–35. 10.1292/jvms.14-0284 25284120 PMC4349535

[29] Mao Y, Lee AW, A novel role for Gab2 in bFGF-mediated cell survival during retinoic acid-induced neuronal differentiation. J Cell Biol 2005; 170: 305–316. 16009726 10.1083/jcb.200505061 PMC2171408

[30] Wang Z, Zhang H, Xu X, Shi H, Yu X, Wang X, et al bFGF inhibits ER stress induced by ischemic oxidative injury via activation of the PI3K/Akt and ERK1/2 pathways. Toxicol Lett. 2012; 212: 137–146. 10.1016/j.toxlet.2012.05.006 22609091

[31] Zhang HY, Zhang X, Wang ZG, Shi HX, Wu FZ, Lin BB, et al Exogenous basic fibroblast growth factor inhibits ER stress-induced apoptosis and improves recovery from spinal cord injury. CNS Neurosci Ther. 2013; 19: 20–29. 10.1111/cns.12013 23082997 PMC6493620

[32] Edamura K, Kuriyama K, Kato K, Nakano R, Teshima K, Asano K, et al Proliferation capacity, neuronal differentiation potency and microstructures after the differentiation of canine bone marrow stromal cells into neurons. J Vet Med Sci. 2012; 74: 923–927. 22333516 10.1292/jvms.11-0388

[33] Edamura K, Kurosawa T, Nakano R, Teshima K, Asano K, Tanaka S, Influence of an autologous serum-supplemented medium on the proliferation and differentiation into neurons of canine bone marrow stromal cells. J Vet Med Sci. 2012; 74: 817–820. 22293468 10.1292/jvms.11-0367

[34] Nakano R, Edamura K, Sugiya H, Narita T, Okabayashi K, Moritomo T, et al Evaluation of mRNA expression levels and electrophysiological function of neuron-like cells derived from canine bone marrow stromal cells. Am J Vet Res. 2013; 74: 1311–1320. 10.2460/ajvr.74.10.1311 24066915

[35] Bradford MM, A rapid and sensitive method for the quantitation of microgram quantities of protein utilizing the principle of protein-dye binding. Anal Biochem. 1976; 7: 248–254. 10.1016/0003-2697(76)90527-3 942051

[36] Niidome T, Nonaka H, Akaike A, Kihara T, Sugimoto H, Basic fibroblast growth factor promotes the generation of microtubule-associated protein 2-positive cells from microglia. Biochem Biophys Res Commun. 2009; 390:1018–1022. 10.1016/j.bbrc.2009.10.100 19854155

[37] Hughes SE, Differential expression of the fibroblast growth factor receptor (FGFR) multigene family in normal human adult tissues. J Histochem Cytochem. 1997; 45: 1005–1019. 9212826 10.1177/002215549704500710

[38] Wang JK, Gao G, Goldfarb M, Fibroblast growth factor receptors have different signaling and mitogenic potentials. Mol Cell Biol. 1994; 14: 181–188. 8264585 10.1128/mcb.14.1.181-188.1994 PMC358368

[39] Manning BD, Cantley LC, AKT/PKB signaling: navigating downstream. Cell. 2007; 129: 1261–1274. 17604717 10.1016/j.cell.2007.06.009 PMC2756685

[40] Ford-Perriss M, Abud H, Murphy M, Fibroblast growth factors in the developing central nervous system. Clin Exp Pharmacol Physiol. 2001; 28: 493–503. 11422214 10.1046/j.1440-1681.2001.03477.x

[41] Itoh N, Ornitz DM, Evolution of the Fgf and Fgfr gene families. Trends Genet. 2004; 20: 563–569. 15475116 10.1016/j.tig.2004.08.007

[42] Zhang F, Clarke JD, Santos-Ruiz L, Ferretti P, Differential regulation of fibroblast growth factor receptors in the regenerating amphibian spinal cord in vivo. Neuroscience. 2002; 114: 837–48. 12379240 10.1016/s0306-4522(02)00321-4

[43] Chen X, Huang J, Li J, Han Y, Wu K, Xu P, Tra2βl regulates P19 neuronal differentiation and the splicing of FGF-2R and GluR-B minigenes. Cell Biol Int. 2004; 28: 791–799. 15563401 10.1016/j.cellbi.2004.07.009

[44] McIntosh I, Bellus GA, Jab EW, The pleiotropic effects of fibroblast growth factor receptors in mammalian development. Cell Struct Funct. 2000; 25: 85–96. 10885578 10.1247/csf.25.85

[45] Khonsari RH, Delezoide AL, Kang W, Hébert JM, Bessières B, Bodiguel V, et al Central nervous system malformations and deformations in FGFR2-related craniosynostosis. Am J Med Genet. A. 2012; 158A: 2797–2806. 10.1002/ajmg.a.35598 22987770

[46] Terada K, Kojima Y, Watanabe T, Izumo N, Chiba K, Karube Y, Inhibition of nerve growth factor-induced neurite outgrowth from PC12 cells by dexamethasone: signaling pathways through the glucocorticoid receptor and phosphorylated Akt and ERK1/2. PLoS One. 2014; 9: e93223, 10.1371/journal.pone.0093223 24667984 PMC3965538

[47] Cosker KE, Eickholt BJ, Phosphoinositide 3-kinase signalling events controlling axonal morphogenesis. Biochem. Soc Trans. 2007; 35: 207–210. 17371239 10.1042/BST0350207

[48] Datta SR, Brunet A, Greenberg ME, Cellular survival: a play in three Akts. Genes Dev. 1999; 13: 2905–2927. 10579998 10.1101/gad.13.22.2905

[49] Zurashvili T, Cordón-Barris L, Ruiz-Babot G, Zhou X, Lizcano JM, Gómez N, et al Interaction of PDK1 with phosphoinositides is essential for neuronal differentiation but dispensable for neuronal survival. Mol Cell Biol. 2013; 33: 1027–1040. 10.1128/MCB.01052-12 23275438 PMC3623085

[50] Brunet A, Datta SR, Greenberg ME, Transcription-dependent and -independent control of neuronal survival by the PI3K-Akt signaling pathway. Curr Opin Neurobiol. 2001; 11: 297–305. 11399427 10.1016/s0959-4388(00)00211-7

[51] Alessi DR, Andjelkovic M, Caudwell B, Cron P, Morrice N, Cohen P, et al Mechanism of activation of protein kinase B by insulin and IGF-1. EMBO J. 1996; 15: 6541–6551. 8978681 PMC452479

[52] Anderson KE, Coadwell J, Stephens LR, Hawkins PT, Translocation of PDK-1 to the plasma membrane is important in allowing PDK-1 to activate protein kinase B. Curr Biol. 1998; 8: 684–691. 9637919 10.1016/s0960-9822(98)70274-x

[53] Read DE, Gorman AM, Involvement of Akt in neurite outgrowth. Cell Mol Life Sci. 2009; 66: 2975–2984. 10.1007/s00018-009-0057-8 19504044 PMC11115732

[54] Park JH, Lee SB, Lee KH, Ahn JY, Nuclear Akt promotes neurite outgrowth in the early stage of neuritogenesis. BMB Rep. 2012; 45: 521–525. 23010173 10.5483/bmbrep.2012.45.9.114

[55] Shi SH, Jan LY, Jan YN, Hippocampal neuronal polarity specified by spatially localized mPar3/mPar6 and PI 3-kinase activity. Cell. 2003; 112: 63–75. 12526794 10.1016/s0092-8674(02)01249-7

[56] Castelo-Branco G, Rawal N, Arenas E, GSK-3β inhibition/β-catenin stabilization in ventral midbrain precursors increases differentiation into dopamine neurons. J Cell Sci. 2004; 117: 5731–5737. 15522889 10.1242/jcs.01505

[57] Logan CY, Nusse R, The Wnt signaling pathway in development and disease. Annu Rev Cell Dev Biol. 2004; 20: 781–810. 15473860 10.1146/annurev.cellbio.20.010403.113126

[58] Zaragosi LE, Wdziekonski B, Fontaine C, Villageois P, Peraldi P, Dani C, Effects of GSK3 inhibitors on in vitro expansion and differentiation of human adipose-derived stem cells into adipocytes. BMC Cell Biol. 2008; 9: 11, 10.1186/1471-2121-9-11 18271953 PMC2257931

[59] Ojeda L, Gao J, Hooten KG, Wang E, Thonhoff JR, Dunn TJ, et al Critical role of PI3K/Akt/GSK3β in motoneuron specification from human neural stem cells in response to FGF2 and EGF. PLoS One. 2011; 6: e23414 10.1371/journal.pone.0023414 21887250 PMC3160859

[60] Dastjerdi FV, Zeynali B, Tafreshi AP, Shahraz A, Chavoshi MS, Najafabadi IK, et al Inhibition of GSK-3β enhances neural differentiation in unrestricted somatic stem cells. Cell Biol Int. 2012; 36: 967–972. 10.1042/CBI20110541 22775567

[61] Yu Q, Liu L, Duan Y, Wang Y, Xuan X, Zhou L, et al Wnt/β-catenin signaling regulates neuronal differentiation of mesenchymal stem cells. Biochem Biophys Res Commun. 2013; 439: 297–302. 10.1016/j.bbrc.2013.08.030 23958304

[62] Tsai HL, Deng WP, Lai WF, Chiu WT, Yang CB, Tsai YH, et al Wnts enhance neurotrophin-induced neuronal differentiation in adult bone-marrow-derived mesenchymal stem cells via canonical and noncanonical signaling pathways. PLoS One. 2014; 9: e104937 10.1371/journal.pone.0104937 25170755 PMC4149376

[63] Han J, Wang B, Xiao Z, Gao Y, Zhao Y, Zhang J, et al Mammalian target of rapamycin (mTOR) is involved in the neuronal differentiation of neural progenitors induced by insulin. Mol Cell Neurosci. 2008; 39: 118–124. 10.1016/j.mcn.2008.06.003 18620060

[64] Park SS, Lee YJ, Han HJ, Kweon OK, Role of laminin-111 in neurotrophin-3 production of canine adipose-derived stem cells: involvement of Akt, mTOR, and p70S6K. J Cell Physiol. 2011; 226: 3251–3260. 10.1002/jcp.22686 21321942

[65] Mruthyunjaya S, Rumma M, Ravibhushan G, Anjali S, Padma S. c-Jun/AP-1 transcription factor regulates laminin-1-induced neurite outgrowth in human bone marrow mesenchymal stem cells: role of multiple signaling pathways. FEBS Lett. 2011: 585:1915–1922. 10.1016/j.febslet.2011.04.072 21570970

[66] Trzaska KA, Kuzhikandathil EV, Rameshwar P, Specification of a dopaminergic phenotype from adult human mesenchymal stem cells. Stem Cells. 2007; 25: 2797–2808. 17656644 10.1634/stemcells.2007-0212

[67] Zhang Z, Alexanian AR, Dopaminergic-like cells from epigenetically reprogrammed mesenchymal stem cells. J Cell Mol Med. 2012; 16: 2708–14. 10.1111/j.1582-4934.2012.01591.x 22681532 PMC4118239

[68] Gong Y, Wang H, Xia H, Stable transfection into rat bone marrow mesenchymal stem cells by lentivirus-mediated NT-3. Mol Med Rep. 2015; 11: 367–73. 10.3892/mmr.2014.2727 25333669

[69] Li L, Li Y, Jiang H, Neurotrophine-3 may contribute to neuronal differentiation of mesenchymal stem cells through the activation of the bone morphogenetic protein pathway. Cell Mol Biol Lett. 2015; 20: 385–403. 10.1515/cmble-2015-0023 26208387

[70] Zeng X, Qiu XC, Ma YH, Duan JJ, Chen YF, Gu HY, Wang JM, Ling EA, Wu JL, Wu W, Zeng YS, Integration of donor mesenchymal stem cell-derived neuron-like cells into host neural network after rat spinal cord transection. Biomaterials. 2015; 53: 184–201. 10.1016/j.biomaterials.2015.02.073 25890718

